# Le schwannome pénien: à propos d'un cas

**DOI:** 10.11604/pamj.2014.18.22.4155

**Published:** 2014-05-06

**Authors:** Younes Essatara, Mohammed Hicham Benazzouz, Rekik Sami, Pierre Bondil

**Affiliations:** 1 Clinique Urologique A, CHU Ibn Sina, Rabat, Maroc; 2 Département d'Urologie-Andrologie, Centre Hospitalier de Chambéry, France

**Keywords:** Schwannome, neurinome, pénis, schwannoma, neurinoma, penis

## Abstract

Le Schwannome, ou neurinome, du pénis est une tumeur extrêmement rare. Il s'agit d'une tumeur de la gaine des nerfs péniens. Un homme de 29 ans opéré pour un neurinome du pénis. La tumeur fut réséquée avec succès tout en préservant la fonction érectile du patient. La résection tumorale est le traitement de choix dans les schwannomes localisés, un suivi régulier est conseillé. Le taux de récidive locale est extrêmement faible après résection complète. A travers cet article, nous allons présenter un nouveau cas de neurinome du pénis et nous allons faire une brève revue de la littérature sur cette pathologie rare.

## Introduction

Le neurinome p énien désigne une tumeur qui provient des cellules de Schwann des nerfs périphériques du pénis [1]. Cette tumeur est très inhabituelle [2] et depuis la première description en 1968 [3], seuls 30 cas de schwannome localisé dans la région du pénis ont été décrits pour des hommes de 10 à 74 ans, avec une prévalence entre 35 et 40 [4, 5]. A travers cet article, nous allons présenter un nouveau cas de neurinome du pénis et nous allons faire une brève revue de la littérature sur cette pathologie rare.

## Patient et observation

**Figure 1:**
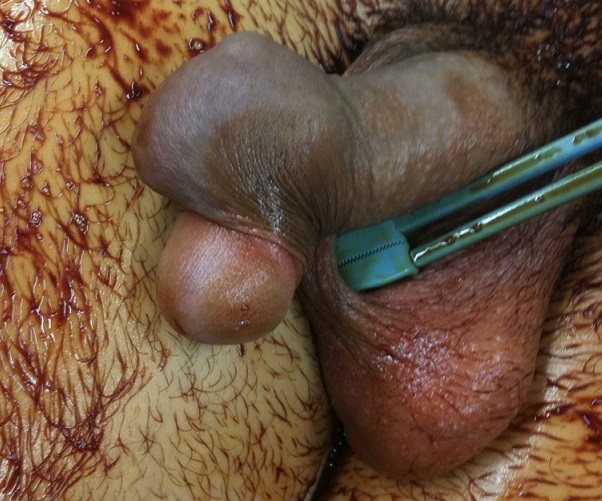
Nodule exophytique, ovoïde et élastique de la face dorsal de la verge

Nous rapportons le cas d´un homme âgé de 29 ans qui s´est présenté dans notre formation pour une tuméfaction du dos de la verge évoluant depuis 10 ans avec une augmentatation progressive de la taille de cette masse durant la derniere année sans aucun autre symptôme associé (Figure 1). Le patient sans notion d´antécédents de maladie systémique ou héréditaire, de traumatisme du pénis, de maladie sexuellement transmissible ou de comportement sexuel anormal ne souffrait d´aucune douleur pendant les érections ou les rapports sexuels ni de troubles de la sensibilité.

L´examen physique avait montré un nodule élastique, mesurant 4 cm de diamètre à la face dorsale du pénis. Le patient a bénéficié d´ une résection chirurgicale du nodule associée à une posthectomie.

Sur l´examen macroscopique, la pièce se présentait sous forme d´un nodule bien limité de 4 cm de diamètre de consistance ferme, recouvert d´un lambeau cutané de prépuce mesurant 5 x 5,5 cm. A la coupe, la tumeur avait une tranche de section polychrome, parsemée de cavités vasculaires.

**Figure 2:**
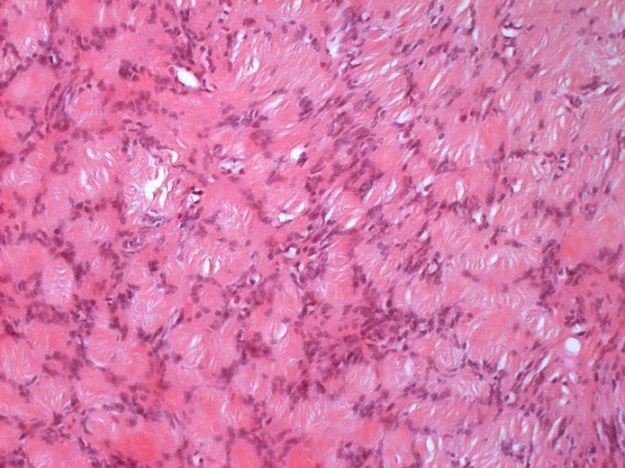
Hématoxyline-éosine: structures palissadiques caractéristiques du type Antoni A avec les nodules de Verocay. Grossissement x 100

L´examen histologique retrouvait une lésion d´architecture nodulaire, limitée par une capsule de tissu conjonctif, constituée de cellules fusiformes à limites cytoplasmiques imprécises, organisées en faisceaux entrelacés tourbillonnant autour d´axes conjonctifs hyalinisés. Par places, ces cellules formaient des structures palissadiques et des nodules de Verocay (Figure 2). Les noyaux étaient le siège d´atypies discrètes; le stroma était plus ou moins abondant, parfois scléro-oedémateux et contenait des cavités vasculaires de taille et de forme variable. Aucune activité mitotique ni nécrose n´était notée. En surface, le prépuce se composait d´un épiderme bien différencié, sous-tendu par un tissu conjonctif lâche et une lame de muscle lisse.

L´étude immunohistochimique était confirmative montrant une positivité de l´anticorps anti PS 100 et de la vimentine. Après un recul de 17 mois, sa fonction érectile est normale. Il n´y a pas de perturbation de la sensibilité ni de signes évidents de récidive locale.

## Discussion

Les schwannomes sont des tumeurs des gaines nerveuses, elles peuvent survenir sporadiquement chez les patients atteints de neurofibromatose et peuvent atteindre n´importe quel partie du corps avec une prédilection pour la tête le cou et la face antérieure des extrémités [6, 7]. Malgré la riche innervation de la région génitale, le schwannome pénien reste une tumeur extrêmement rare [2] et doit être inclus dans le diagnostic différentiel des tumeurs du pénis.

La plupart des schwannomes du pénis sont des tumeurs bénignes, unifocales et asymptomatiques [2]. Certains patients se plaignent de troubles lors des rapports sexuels [8]. La douleur causée par ces nodules du pénis peut également être lié à l´effet de compartiment, en particulier lors des érections [4]. Dans ces conditions, l´excision chirurgicale est indiquée [8].

L´échographie ne peut donner plus d´informations sur les caractéristiques des tissus à la différence de l´imagerie par IRM [9]. En outre, la dépression de l´albuginée dans les images IRM donne une idée sur la localisation de la tumeur [4]. Chez notre patient, aucune imagerie préopératoire n´a été réalisée.

La Résection locale est le traitement de choix pour les schwannomes localisés, et un suivi régulier est conseillé [7, 8]. Le taux de récidive locale est extrêmement faible après résection complète [10]. La récidive peut survenir en cas d´éxérèse incomplète [11]. Notre patient a bénéficié d´ une résection complète sans aucun signe de récidive après un recul de 17 mois.

Histologiquement, il existe deux types d´architecture. Des zones compactes de cellules fusiformes, avec un cytoplasme éosinophile, disposées en palissades (nodules Verocay) et en rouleaux, correspondant à des zones d´Antoni A. Des Secteurs de cellules tumorales rares dans un tissus myxoide appelées zones d´Antoni B [7].

A l´examen immuno-histochimique, le marquage est positif à la protéine S-100, montrant l´origine nerveuse de la tumeur [8].

## Conclusion

Les schwannomes péniens sont des tumeurs généralement bénignes et habituellement asymptomatiques. Par conséquent, l'indication opératoire n'est pas toujours formelle. En l'absence de manifestations cliniques caractéristiques, le diagnostic final repose sur l’étude anatomo-pathologique postopératoire. L'excision simple et la surveillance restent le traitement de choix.
